# TRIM11 Negatively Regulates IFNβ Production and Antiviral Activity by Targeting TBK1

**DOI:** 10.1371/journal.pone.0063255

**Published:** 2013-05-13

**Authors:** Younglang Lee, Byeongwoon Song, Chankyu Park, Ki-Sun Kwon

**Affiliations:** 1 Department of Biological Sciences, Korea Advanced Institute of Science and Technology, Daejeon, Korea; 2 Laboratory of Cell Signaling, Aging Research Center, Korea Research Institute of Bioscience and Biotechnology, Daejeon, Korea; 3 Department of Molecular and Cellular Biology, University of California Davis, Davis, California, United States of America; Johns Hopkins School of Medicine, United States of America

## Abstract

The innate immune response is a host defense mechanism against infection by viruses and bacteria. Type I interferons (IFNα/β) play a crucial role in innate immunity. If not tightly regulated under normal conditions and during immune responses, IFN production can become aberrant, leading to inflammatory and autoimmune diseases. In this study, we identified TRIM11 (tripartite motif containing 11) as a novel negative regulator of IFNβ production. Ectopic expression of TRIM11 decreased IFNβ promoter activity induced by poly (I:C) stimulation or overexpression of RIG-I (retinoic acid-inducible gene-I) signaling cascade components RIG-IN (constitutively active form of RIG-I), MAVS (mitochondrial antiviral signaling protein), or TBK1 (TANK-binding kinase-1). Conversely, TRIM11 knockdown enhanced IFNβ promoter activity induced by these stimuli. Moreover, TRIM11 overexpression inhibited the phosphorylation and dimerization of IRF3 and expression of IFNβ mRNA. By contrast, TRIM11 knockdown increased the IRF3 phosphorylation and IFNβ mRNA expression. We also found that TRIM11 and TBK1, a key kinase that phosphorylates IRF3 in the RIG-I pathway, interacted with each other through CC and CC2 domain, respectively. This interaction was enhanced in the presence of the TBK1 adaptor proteins, NAP1 (NF-κB activating kinase-associated protein-1), SINTBAD (similar to NAP1 TBK1 adaptor) or TANK (TRAF family member-associated NF-κB activator). Consistent with its inhibitory role in RIG-I-mediated IFNβ signaling, TRIM11 overexpression enhanced viral infectivity, whereas TRIM11 knockdown produced the opposite effect. Collectively, our results suggest that TRIM11 inhibits RIG-I-mediated IFNβ production by targeting the TBK1 signaling complex.

## Introduction

The innate immune system is the first line of host defense against invading pathogens [1]. The innate immune response is initiated when pattern recognition receptors (PRRs) such as Toll-like receptors and RIG-I-like receptors sense pathogen-derived molecules, known as pathogen associated molecular patterns (PAMPs) [2], [3]. Signaling pathways activated downstream of PRRs lead to activation of transcription factors such as NF-κB, AP-1 (activator protein-1) and interferon regulatory factors (IRFs) that induce the expression of pro-inflammatory cytokines and type-I interferons (IFNs). IRF3, in particular, is the key transcription factor of type I IFN gene expression during viral infection [4], [5].

TBK1 (TANK binding kinase-1), initially identified as a protein kinase that interacts with TANK (TRAF family member-associated NF-κB activator), was subsequently shown to act as an IKK (IκB kinase)-activating kinase responsible for NF-κB activation in response to growth factors [6], [7]. Recent studies have reported a new function of TBK1 as a virus-activated kinase necessary for IRF3 activation and establishment of an antiviral state [8], [9]. Aberrant production of IFNβ and inflammatory cytokines can cause inflammatory and autoimmune diseases; thus, the activity of TBK1 is tightly regulated [10]. Several molecules have been shown to positively or negatively regulate IFNβ production through effects on TBK1 or other components of the RIG-I pathway. Hsp90 maintains stability of TBK1 and facilitates signal transduction through formation of a complex with TBK1 and IRF3 [11]. Nrdp1 (neuregulin receptor degradation protein-1) enhances TBK1 activity by catalyzing Lys63-linked polyubiquitination of TBK1 [12]. GSK3β (glycogen synthase kinase 3 beta) positively regulate IFNβ production by promoting TBK1 self-activation [13]. PSMA7 (proteasome subunit alpha type-7) interacts with MAVS and negatively regulates by inducing its proteasome-dependent degradation [14]. TAX1BP1 (tax1-binding protein-1) and zinc finger protein A20 (also known as tumor necrosis factor alpha-induced protein 3) terminate antiviral signaling by disrupting Lys63-linked polyubiquitination of TBK1 and IKKε (inducible IKK) [15]. NLRP4 (NLR family pyrin domain-containing 4) promotes degradation of TBK1 by recruiting the E3 ligase DTX4 (deltex 4 homolog) to TBK1 and promoting Lys48-linked polyubiquitination of TBK1 [16]. TRIP (TRAF-interacting protein) negatively regulates the production of IFNβ by promoting TBK1 degradation through Lys48-linked polyubiquitination [17]. RNF11 impedes antiviral signaling by inhibiting Lys63-linked polyubiquitination of TBK1 [18].

The TRIM proteins are members of a large family of proteins characterized by their shared tripartite motif structure, also known as the RBCC (RING finger, B-box, and coiled-coil) domain [19]. TRIM proteins are involved in diverse cellular processes, including cell proliferation, differentiation, oncogenesis, and apoptosis [20]. It has recently been reported that some TRIM proteins are involved as regulators in the immune system, but their precise mechanisms of action are not yet fully understood [21]–[24].

TRIM11 contains the RBCC domain and a C-terminal B30.2/SPRY domain. To date, the following substrates of TRIM11 for ubiquitin mediated degradation have been identified: Humanin, 24-amino-acid neuroprotective peptide; activator-recruited cofactor 105-kDa component (ARC105), a component of the ARC complex that mediates chromatin-directed transcriptional activation; Pax6, a member of the paired-box family of transcription factors; and PHOX2B, a paired box homeodomain transcription factor [25]–[28]. In addition, it has been reported that TRIM11 acts in a RING domain-dependent manner to reduce the levels of TRIM5α protein, an inhibitor of HIV infection [29].

In this study, we demonstrate that TRIM11 interacts with TBK1, a key component of RIG-I-mediated IFNβ signaling and inhibits IRF3 activation and IFNβ mRNA expression, reducing the IFN-induced antiviral state against HSV-1 and VSV-GFP (Vesicular stomatitis virus encoding green fluorescent protein). Collectively, our results suggest a new role for TRIM11 in innate immunity.

## Materials and Methods

### Plasmids and Viruses

HA- and mCherry-tagged TRIM11 expression vectors were generated by amplifying full-length mouse TRIM11 cDNA (IMAGE clone M4014766) by polymerase chain reaction (PCR) and cloning into the HA tagging pcDNA3 vector and mCherry tagging pLentiM1.4 vector, respectively. HA-tagged deletion mutants of TRIM11 lacking the CC and B30.2/SPRY domain (amino acid residues 128–483) and B30.2/SPRY domain only (amino acid residues 283–483) were cloned into HA tagging pcDNA3 vector. FLAG-MAVS and FLAG-TBK1 plasmids were kindly provided by Dr. Glen Barber (University of Miami School of Medicine and Sylvester Comprehensive Cancer Center). Full-length Myc-mTBK1 (Myc-tagged mouse TBK1) and deletion mutants lacking the ubiquitin-like domain (ΔULD), coiled-coil domain 1 (ΔCC1), or coiled-coil domain 2 (ΔCC2) were kindly provided by Dr. Giulio Superti-Furga (Austrian Academy of Sciences). Yellow fluorescent protein (YFP) fusion constructs of NAP1, SINTBAD and TANK were kindly provided by Dr. Felix Randow (University of Cambridge). The YFP-TBK1 expression vector was obtained by cloning TBK1-containing sequences into a YFP tagging pLentiM1.4 vector. FLAG-RIG-IN was gift from Dr. Adolfo García-Sastre (Mount Sinai School of Medicine). GFP-IRF3 was kindly provided by Dr. Joo Young Lee (Gwangju Institute of Science and Technology). IKKε cDNA was provided by Dr. Tom Maniatis (Columbia University) and subcloned into the N-terminal pFLAG-CMV vector (Sigma). An IFNβ-luciferase reporter (IFNβ-Luc) plasmid was kindly provided by Dr. Takashi Fujita (Osaka University). NF-κB-Luc and pCMV-β-gal (CMV-β-gal) plasmid were purchased from Upstate Biotechnology and Clontech, respectively. Herpes simplex virus type 1 (HSV-1, KOS strain) was kindly provided by Dr. Inpyo Choi (Korea Research Institute of Bioscience and Biotechnology). VSV-GFP was kindly provided by Dr. Jae Ung Jung (University of Southern California).

### Cell Culture and Transfection

Human embryonic kidney 293T and Vero (green monkey kidney) cells were grown at 37°C in DMEM (Invitrogen) containing 10% heat-inactivated FBS (JRS), 100 units/ml penicillin, and 100 mg/ml streptomycin (Invitrogen) in a 5% CO_2_ atmosphere. Cells were transfected using calcium phosphate precipitation or polyethylenimine methods or the commercial transfection reagent, Lipofectamine (Invitrogen). Cells were harvested 36 h (or at the indicated times) after transfection.

### Immunoblotting and Antibodies

Cells were lysed in a lysis buffer containing 20 mM HEPES (pH 7.2), 150 mM NaCl, 0.5% Triton X-100, 0.1 mM Na_3_VO_4_, 1 mM NaF, 1 mM 4-(2-aminoethyl)-benzenesulfonyl fluoride hydrochloride (AEBSF), and 5 mg/ml aprotinin. Soluble proteins in cell lysates were separated by sodium dodecyl sulfate-polyacrylamide gel electrophoresis (SDS-PAGE) and analyzed by immunoblotting using anti-FLAG (Sigma), anti-HA (Sigma), anti-Myc (Invitrogen), anti-GFP (Santa Cruz Biotechnology), anti-TBK1 (Santa Cruz Biotechnology and Abcam), anti-IRF3 (Cell Signaling Technology), and anti-phospho-IRF3 (Cell Signaling Technology) antibodies.

### Native Gel Electrophoresis

Native PAGE was performed as previously described [30], with minor modifications. Briefly, 7.5% acrylamide gels (without SDS) were prerun for 30 min at 40 mA in 25 mM Tris and 192 mM glycine (pH 8.4) buffer containing 0.5% deoxycholate in the cathode chamber. Cell lysates in the native sample buffer (62.5 mM Tris–HCl, pH 6.8, 15% glycerol, bromophenol blue) were subjected to electrophoresis for 80 min at 25 mA. Gels were soaked in SDS running buffer (25 mM Tris, pH 8.4, 250 mM glycine, 0.1% SDS) for 10 min and then immunoblotted.

### Immunoprecipitation

Lysates were incubated with anti-FLAG agarose (Sigma), anti-HA agarose (Sigma) or anti-TBK1 antibody (Abcam) at 4°C for several hours, after which the beads were washed three times with cell lysis buffer. The beads were resuspended in 1X SDS-PAGE sample buffer and subjected to SDS-PAGE.

### Luciferase Reporter Assay

Luciferase activity was assessed using the Luciferase Assay System (Promega), according to the manufacturer’s instructions. Luciferase activity was normalized to β-galactosidase activity to adjust for transfection efficiency. Exogenous RIG-IN, MAVS, TBK1, IKKε, and poly (I:C) (10 µg/ml, Sigma) were used as agonists.

### Knockdown of TRIM11

To establish a stable TRIM11 knockdown cell line, small hairpin RNA (shRNA) against human TRIM11 in pLKO.1-puro lentiviral vectors were purchased from Sigma (clone ID NM_145214.2-1530s1c1 and NM_145214.2-1147s1c1). shRNA lentiviral particles were generated in 293T cells by transient transfection with pLP1, pLP2, pVSV-G (Invitrogen) and shRNA lentiviral vector or pLKO.1-scrambled (control) vector (SHC002V; Sigma) using Lipofectamine (Invitrogen). Forty-eight hours after transfection, supernatants containing lentiviral particles were collected and used to infect 293T cells in the presence of 4 µg/ml polybrene. Infected cells were selected by incubation with 2 µg/ml puromycin for 2–3 weeks, and used in experiments as indicated. For transient knockdown experiments, TRIM11-specific siRNA (5′- GUCUGUUCAGCAGGUGUGU-3′) and control siRNA were purchased from Bioneer. 293T cells were transfected with the above siRNAs using the Lipofectamine (Invitrogen).

### RT-PCR Analysis

Total RNA was extracted using Easy-Blue reagent (iNtRON Biotechnology), according to the manufacturer’s instructions, and treated with RNase-free DNase I (Takara) to remove contaminating genomic DNA. cDNA was synthesized from total RNA by reverse transcription (RT) using a DiaStar RT kit (Solgent). PCR was performed using specific primers targeting IFNβ, TRIM11, TBK1, or GAPDH genes. Quantitative RT-PCR analysis was performed with the primer targeting TRIM11 using the Step One Plus real-time PCR System (Applied Biosystems). Data were normalized by the abundance of β-actin mRNA.

### Subcellular Localization Analysis

293T cells were cultured on coverslips and cotransfected with mCherry-TRIM11 and YFP-TBK1 plasmids using the calcium phosphate precipitation method. After 36 h, cells were fixed with 3.7% paraformaldehyde for 10 min. and nuclei were stained with 4,6-diamidino-2-phenylindole (DAPI). The localization of both proteins was monitored by confocal microscopy (Olympus FluoView FV1000).

### Plaque-reduction Assay

Plaque-reduction assays were performed as previously described [31]–[33], with modifications. Briefly, 293T cells were transfected with the indicated DNAs. After 12 h, the medium was removed and replaced with fresh DMEM. The medium was collected 12 h later. Vero cells were seeded in 12-well plates at a density of 3×10^5^ cells/well, and then 6 h later, were pretreated with the collected medium. After 12 h, Vero cells were infected with HSV-1 virus for 1 h, and then overlaid with methylcellulose overlay medium, adding the same collected medium used for pretreatment. After 3–4 days of incubation, plates were stained with crystal violet and plaques were counted.

### VSV-GFP Infection Assay

Supernatants were obtained as described above for the plaque-reduction assay. Vero cells were seeded in 12-well plates at a density of 0.8×10^5^ cells/well, and then 6 h later were pretreated with the collected medium. After 12 h, Vero cells were infected with VSV-GFP virus (10 plaque forming units [PFU]/cell) for 1 h. The medium was then replaced with fresh DMEM, adding the same collected medium used for pretreatment. GFP expression was analyzed by fluorescence microscopy at the indicated times.

## Results

### TRIM11 is a Negative Regulator of Innate Immunity

**Figure 1:**
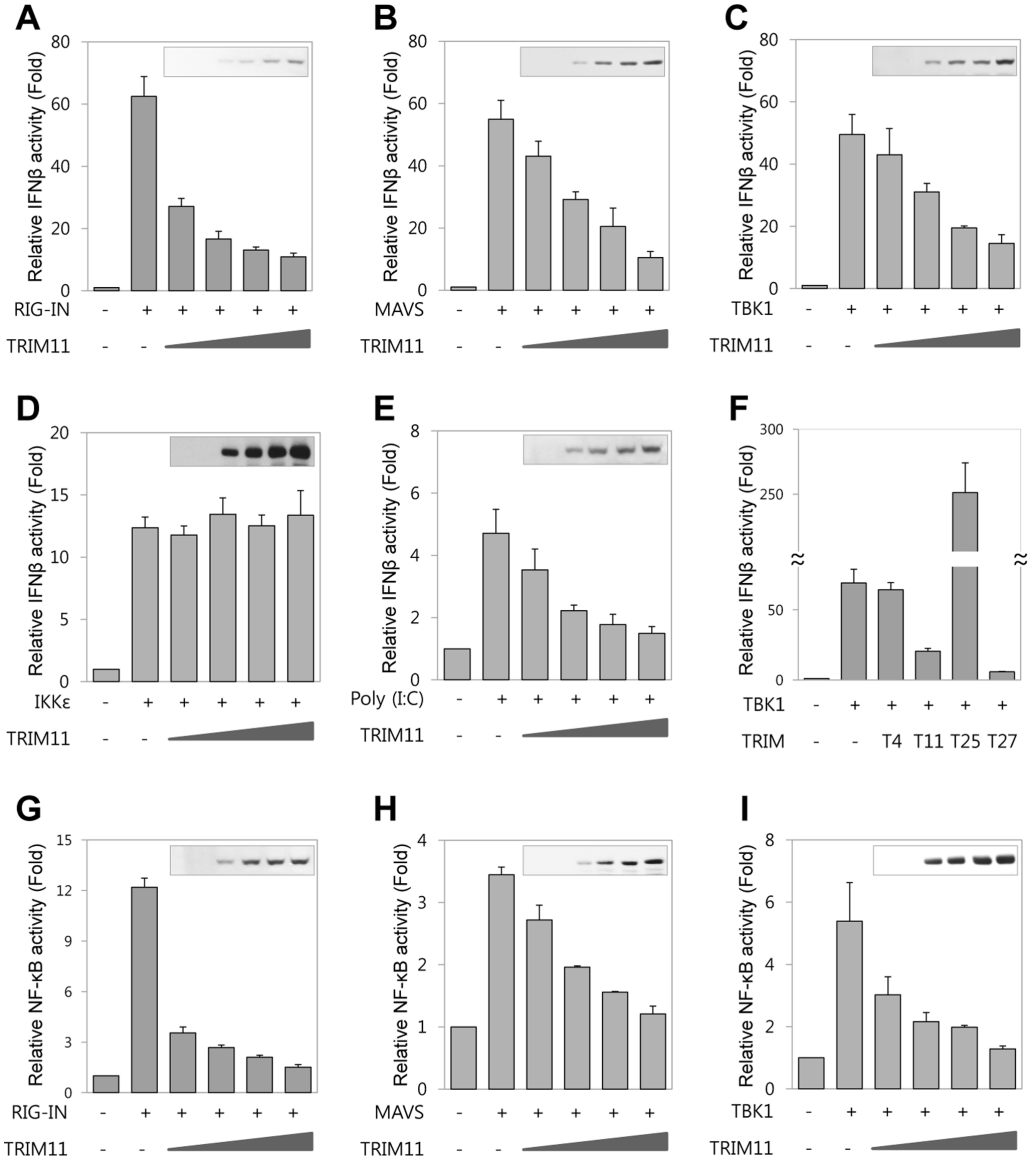
Effect of TRIM11 expression on IFNβ and NF-κB promoter activity. 293T cells were transfected with IFNβ-Luc (**A–F**) or NF-κB-Luc (**G–I**) together with CMV-β-gal plasmid and increasing amount of HA-TRIM11 plasmid or the indicated TRIM plasmids. Twenty-four hours after transfection, cells were further transfected with poly (I:C) (10 µg; **E**) for 12 h. Expression plasmids for RIG-IN (**A, G**), MAVS (**B, H**) or TBK1 (**C, F, I**), or IKKε (**D**) were included in the initial transfection, as indicated. Luciferase activity was measured and normalized for transfection efficiency using β-galactosidase activity. Results are mean values from three independent experiments. Error bar represents SD. Expression levels of HA-TRIM11 were assessed by anti-HA immunoblotting (inset).

**Figure 2:**
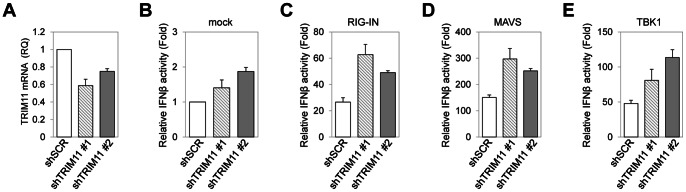
Knockdown of TRIM11 enhances IFNβ promoter activity. (**A**) TRIM11 expression in 293T cells stably expressing TRIM11-specific shRNA (shTRIM11 #1 and shTRIM11 #2) or scrambled shRNA (shSCR) was analyzed by quantitative RT-PCR and normalized to β-actin. (**B–E**) 293T cells stably expressing shTRIM11 #1, shTRIM11 #2 or shSCR were transfected with IFNβ-Luc and CMV-β-gal plasmid together with RIG-IN (**C**), MAVS (**D**) or TBK1 (**E**) expression plasmid. Luciferase activity was measured and normalized for transfection efficiency using β-galactosidase activity. Results are representative of three independent experiments. Error bar represents SD.

In order to identify new TRIM members that play a role in innate immunity, we examined the effect of expression of twenty-eight TRIM proteins on IFNβ expression using an IFNβ promoter activity assay. In addition, we performed co-immunoprecipitation assay to test whether TRIM proteins interact with components of RIG-I signaling pathway such as RIG-I, TBK1 and IRF3. From these experiments, we identified several TRIM proteins having distinct effects on IFNβ promoter activity and interacting with RIG-I, TBK1 or IRF3 (unpublished data). Among these, we chose TRIM11 for further analysis, since TRIM11 efficiently decreased the IFNβ promoter activity and interacted with TBK1 (see below). To test the role of TRIM11 in RIG-I signaling-mediated innate immunity, we measured IFNβ and NF-κB promoter activity, both of which are transcriptionally controlled by RIG-I signaling [34]. Ectopic expression of RIG-IN (constitutively active form of RIG-I lacking the C-terminus), MAVS or TBK1 increased both IFNβ and NF-κB promoter activity (see the fold increases in Figure 1). Coexpression of TRIM11 inhibited both IFNβ (Fig. 1A–C) and NF-κB (Fig. 1G–I) promoter activity in a dose dependent manner. However, TRIM11 coexpression did not affect IFNβ promoter activity induced by IKKε, which, like TBK1, is an IKK-related kinase (Fig. 1D). We next investigated whether TRIM11 reduced IFNβ production induced by poly (I:C), which mimics an RNA virus. Overexpression of TRIM11 markedly reduced poly (I:C)-induced IFNβ promoter activity (Fig. 1E). To compare the effect of TRIM11 on the regulation of IFNβ production with that of other TRIM proteins, we performed IFNβ reporter assays using TRIM4, TRIM25, and TRIM27. TRIM4 is a close relative of TRIM11 [35], whereas TRIM25 is a positive regulator of the RIG-I signaling pathway [36] and TRIM27 is known to inhibit IKKs [37]. TRIM4 did not affect IFNβ promoter activity; however, TRIM27 inhibited IFNβ promoter activity to the same extent as TRIM11, and TRIM25 dramatically enhanced IFNβ promoter activity (Fig. 1F), as expected. To examine whether TRIM11 activity is specific for IFNβ and NF-κB promoters, we performed reporter assays using TOP-flash as a non-relevant promoter. We found that overexpression of TRIM11 had no effect on the TOP-flash promoter activity induced by active β-catenin (Figure S1). To confirm the negative regulation of IFNβ production by TRIM11, we prepared TRIM11-knockdown 293T cells by infection with lentivirus encoding one of two different shRNAs against TRIM11 and isolated stably infected cells by puromycin selection. Both knockdown cells showed 30% ∼ 40% lower TRIM11 expression levels compared to scrambled shRNA-infected control cells (Fig. 2A). TRIM11 knockdown enhanced the IFNβ promoter activity induced by RIG-IN, MAVS, or TBK1 (Fig. 2B–E). From these results, we conclude that TRIM11 functions as a negative regulator of the RIG-I signaling pathway.

### TRIM11 Negatively Regulates IRF3 Activation

**Figure 3:**
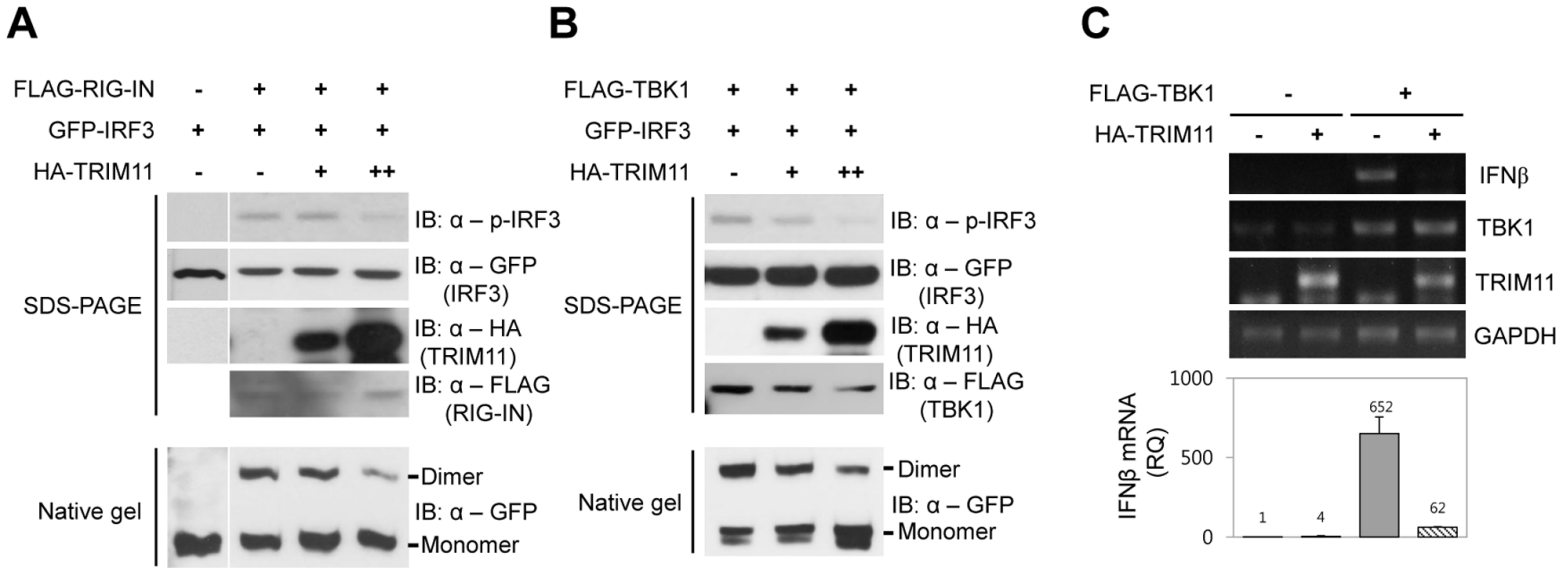
TRIM11 reduces IRF3 activation and IFNβ production. 293T cells were transiently cotransfected with GFP-IRF3 plasmid, increasing amount of HA-TRIM11 plasmid, and FLAG-RIG-IN (**A**) or FLAG-TBK1 (**B**) plasmid. After 36 h, cell extracts were prepared and analyzed by immunoblotting with anti-phospho-IRF3, anti-GFP, anti-FLAG and anti-HA antibodies. The dimerization state of IRF3 was analyzed using native PAGE. (**C**) 293T cells were transfected with FLAG-TBK1 plasmid together with HA-TRIM11 or empty vector. After 36 h, total RNA was extracted and treated with DNase I. The expression of IFNβ, TBK1, TRIM11, and GAPDH was analyzed by RT-PCR (upper panel). Relative quantity (RQ) of IFNβ mRNA was measured by quantitative RT-PCR (lower panel).

**Figure 4:**
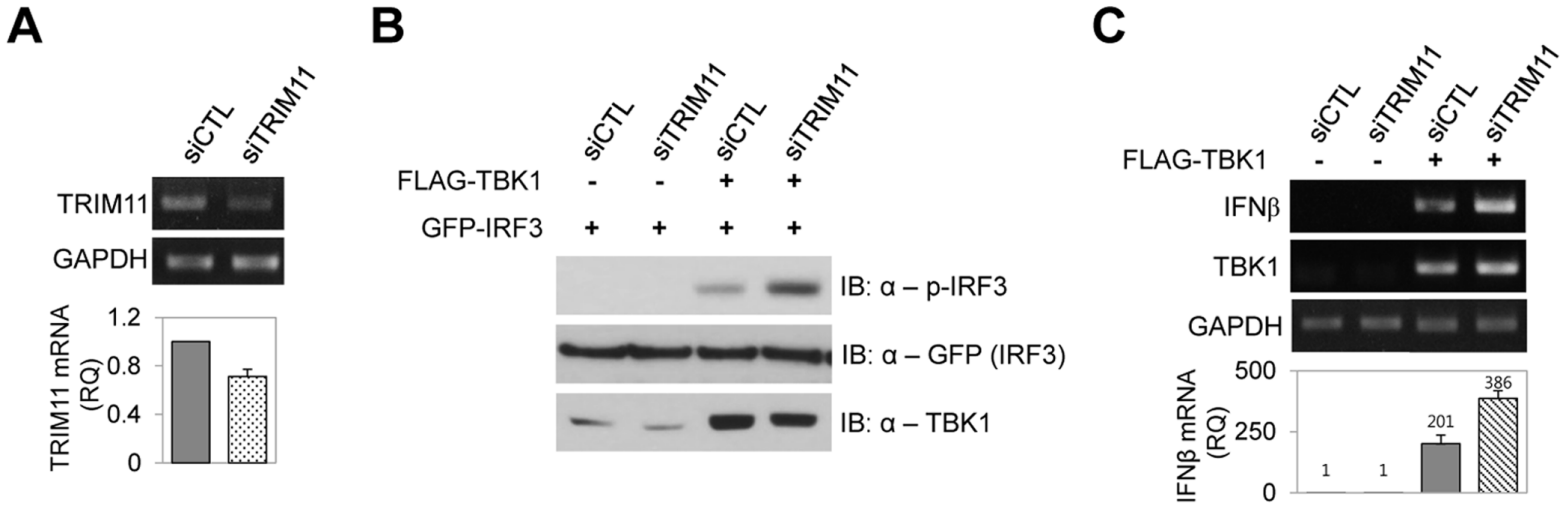
Knockdown of TRIM11 increases IRF3 phosphorylation and IFNβ production. (**A**) TRIM11 expression was RT-PCR (upper panel) and quantitative RT-PCR (lower panel) analyzed at 36 h after siRNA (siCTL or siTRIM11) transfection. (**B**) 293T cells were cotransfected with GFP-IRF3 plasmid, FLAG-TBK1 plasmid and siCTL or siTRIM11, as indicated. After 36 h, cell extracts were prepared and analyzed by immunoblotting with anti-phospho-IRF3, anti-GFP and anti-TBK1 antibodies. (**C**) 293T cells were cotransfected with FLAG-TBK1 plasmid and siCTL or siTRIM11. After 36 h, total RNA was extracted and treated with DNase I. The expression of IFNβ, TBK1 and GAPDH were analyzed by RT-PCR (upper panel). Relative quantity (RQ) of IFNβ mRNA was measured by quantitative RT-PCR (lower panel).

IRF3 is the key transcription factor in the RIG-I pathway responsible for promoting the expression of IFNβ. RIG-I activation leads to the TBK1-dependent phosphorylation of IRF3, resulting in IRF3 dimerization and subsequent nuclear translocation and binding to the IFNβ promoter [38]. PAGE analysis of the level of phosphorylated and dimeric forms of IRF3 showed that both phosphorylation and dimerization of IRF3, induced by either RIG-IN or TBK1 expression, were inhibited by TRIM11 coexpression in a dose-dependent manner (Fig. 3A and B). We then asked whether TRIM11 ultimately inhibits IFNβ expression. Ectopic expression of TRIM11 remarkably reduced the increase in IFNβ mRNA levels induced by TBK1 expression (Fig. 3C). We next investigated the effect of TRIM11 knockdown on IRF3 phosphorylation and IFNβ mRNA expression (Fig. 4). In contrast to the above results, TRIM11 knockdown increased both IRF3 phosphorylation and IFNβ gene expression induced by TBK1 (Fig. 4B and C). Taken together, these data indicate that TRIM11 negatively regulates IRF3 activation and subsequent expression of IFNβ via the RIG-I signaling pathway.

### TRIM11 Interacts with TBK1

**Figure 5:**
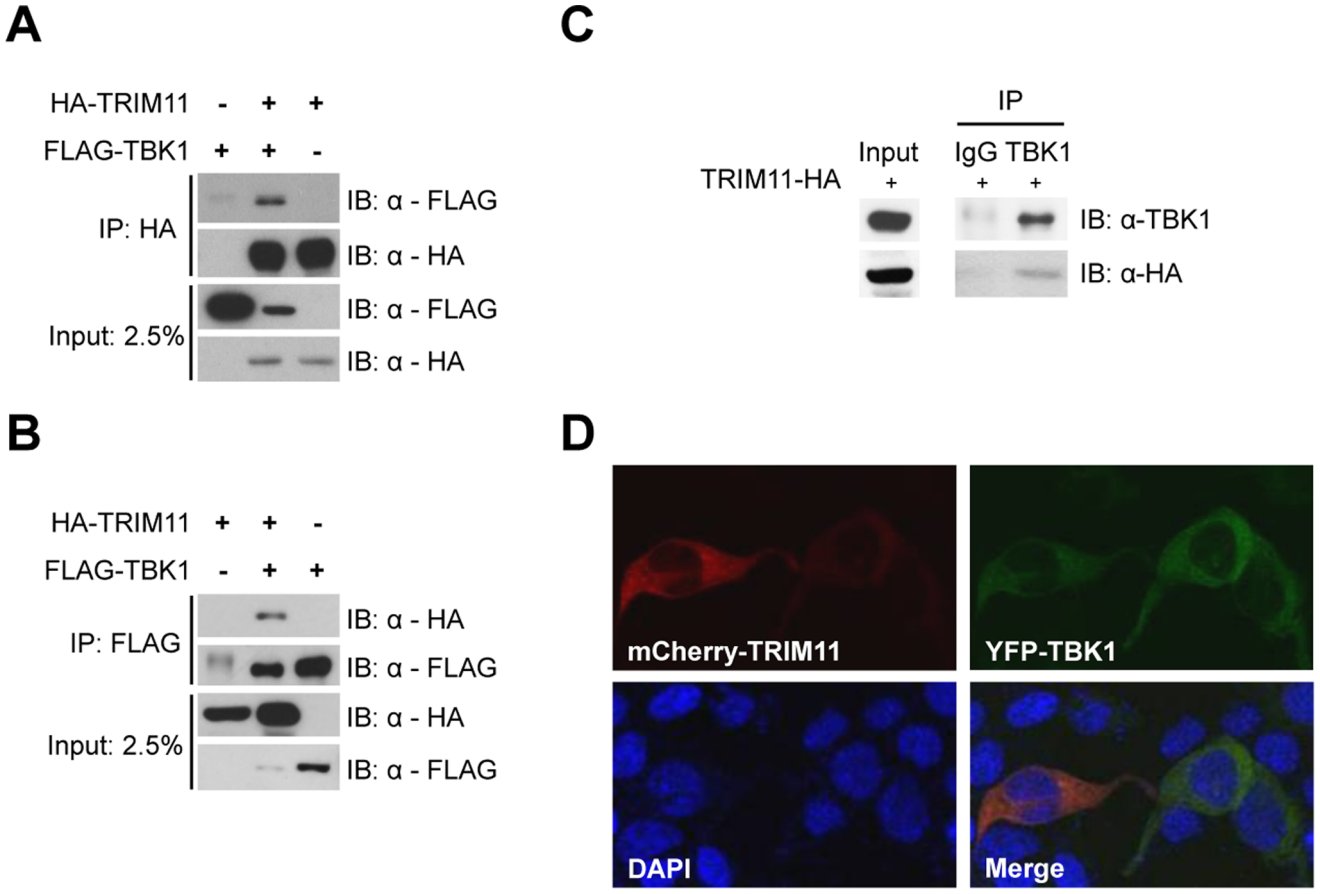
TRIM11 interacts with TBK1. 293T cells were cotransfected with HA-TRIM11 and FLAG-TBK1 plasmids, as indicated. After 36 h, cells were lysed and immunoprecipitated (IP) with anti-HA (**A**) or anti-FLAG agarose (**B**). Immunoblotting (IB) was performed with anti-FLAG and anti-HA antibodies. (**C**) 293T cells were transfected with HA-TRIM11. At 36 hours post-transfection, the cells were lysed and immunoprecipitated with anti-TBK1 antibody or normal rabbit IgG. Immunoblotting was performed with anti-TBK1 and anti-HA antibodies. (**D**) 293T cells were cotransfected with mCherry-TRIM11 and YFP-TBK1 plasmids. After 36 h, TRIM11 and TBK1 expression were monitored by confocal microscopy. Nuclei are shown by DAPI staining.

To confirm the interaction between TRIM11 and TBK1, we performed a reciprocal immunoprecipitation assay using 293T cells cotransfected with HA-TRIM11 and FLAG-TBK1. HA-TRIM11 and FLAG-TBK1 were immunoprecipitated by anti-HA agarose and anti-FLAG agarose, respectively, and the immunoprecipitates were immunoblotted with anti-HA and anti-FLAG antibodies. Consistently, TBK1 was co-precipitated with TRIM11 (Fig. 5A and B). Because tests showed that two commercially available TRIM11 antibodies were ineffective, we examined endogenous TBK1 interactions using the cells in which HA-TRIM11 was transiently expressed. Anti-TBK1 antibody immunoprecipitates, but not rabbit IgG immunoprecipitates, showed coprecipitation of HA-TRIM11 (Fig. 5C), indicating that TRIM11 interacts with the endogenous TBK1 protein. On the basis of the results of these immunoprecipitation assays and IFNβ promoter activity assays, described above, we hypothesize that TRIM11 functions in regulating IRF3 activation at the level of TBK1. Next, we examined the subcellular localization of TRIM11 and TBK1 in 293T cells. Confocal microscopy showed that, in 293T cells transiently cotransfected with mCherry-TRIM11 and YFP-TBK1, both proteins were localized in the cytosol (Fig. 5D).

### TRIM11 Interacts with and Inhibits the Activity of TBK1-adaptor Complexes

**Figure 6:**
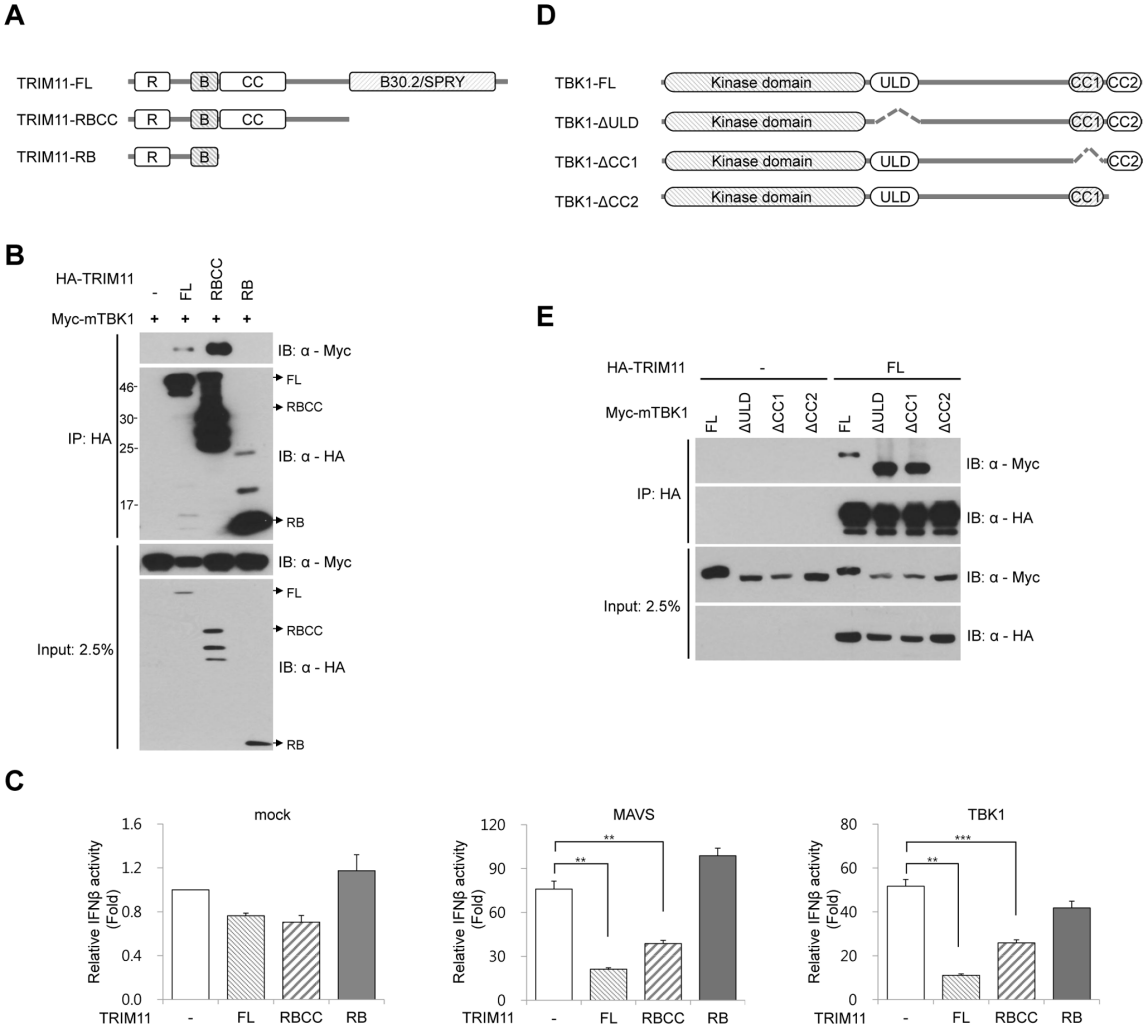
Interaction between TRIM11 and TBK1 is mediated by CC domains. (**A**) Schematic representation of TRIM11-FL and deletion mutants. (**B**) 293T cells were transfected with Myc-mTBK1 plasmid and HA-tagged TRIM11 (FL, RBCC or RB), as indicated. After 36 h, cells were lysed and immunoprecipitated with anti-HA agarose. Immunoblotting (IB) was performed with anti-Myc and anti-HA antibodies. (**C**) 293T cells were cotransfected with HA-tagged TRIM11 (FL, RBCC or RC) and empty vector (left panel), MAVS (middle panel), or TBK1 (right panel) plasmid along with IFNβ-Luc and CMV-β-gal plasmid. After 36 h, luciferase activity was measured and normalized for transfection efficiency using β-galactosidase activity. Results are mean values from three independent experiments. Error bar represents SD. (**D**) Schematic representation of TBK1-FL and domain-deleted mutants. (**E**) 293T cells were cotransfected with Myc-mTBK1 (FL, ΔULD, ΔCC1, or ΔCC2) and HA-TRIM11 plasmids as indicated. After 36 h, cells were lysed and immunoprecipitated with anti-HA agarose. Immunoblotting was performed with anti-Myc and anti-HA antibodies. **P<0.01, ***P<0.001.

**Figure 7:**
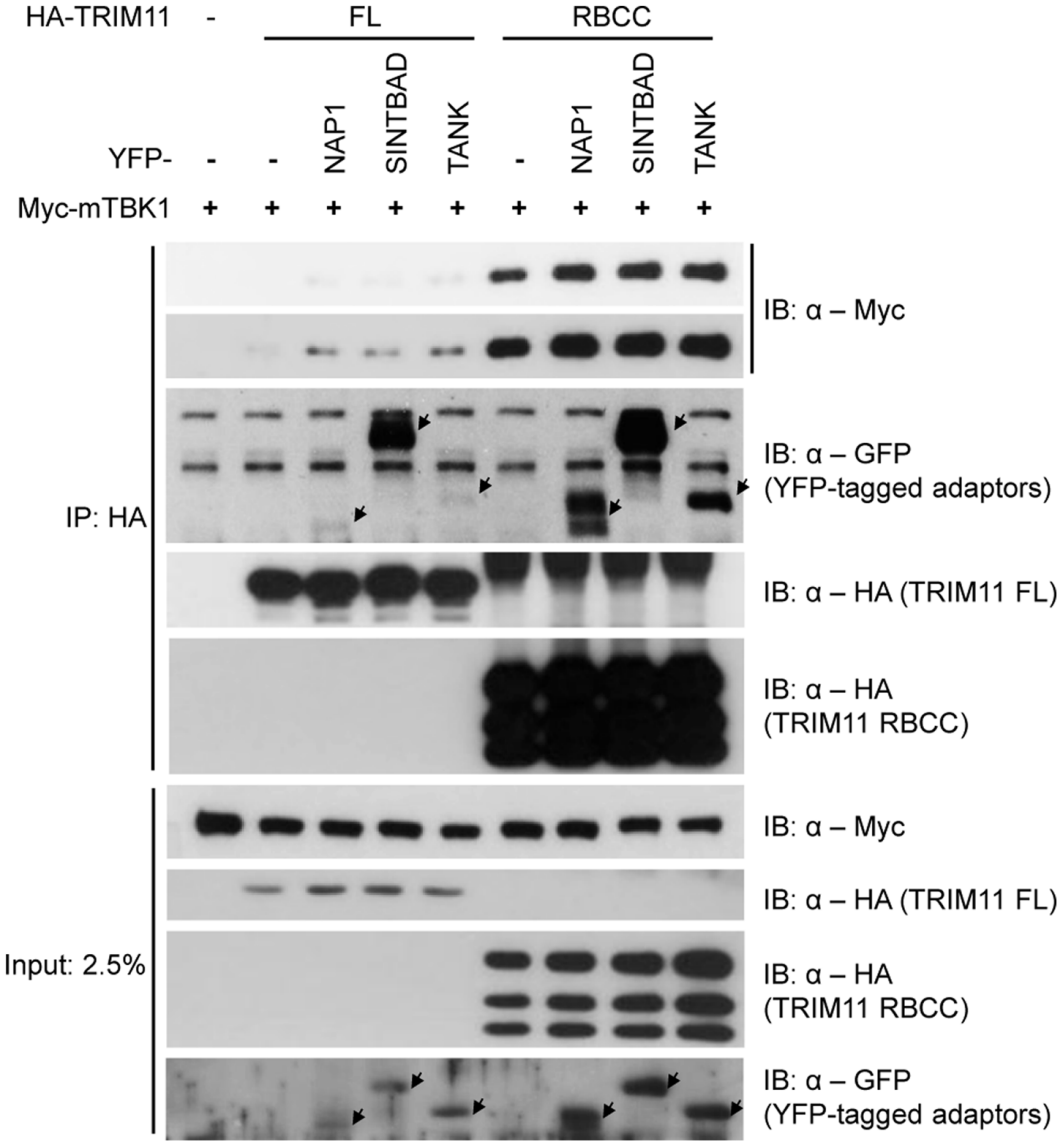
TRIM11 binds strongly to TBK1 associated with adaptor proteins. 293T cells were transfected with HA-TRIM11 (FL or RBCC) and YFP-tagged TBK1 adaptor (NAP1, SINTBAD or TANK) together with Myc-mTBK1 expression plasmids, as indicated. After 36 h, cells were lysed and immunoprecipitated with anti-HA agarose. Immunoblotting was performed with anti-Myc, anti-HA, and anti-GFP antibodies.

To identify the region of TRIM11 that mediates the interaction with TBK1, we constructed two TRIM11 domain-deletion mutants, one lacking the B30.2/SPRY domain but retaining the RBCC domain (TRIM11-RBCC) and the other lacking both CC and B30.2/SPRY domains but retaining RING finger and B-box motifs (TRIM11-RB) (Fig. 6A). We then analyzed the interactions of these two deletion constructs of TRIM11 as well as that of a full-length (TRIM11-FL) construct with Myc-mTBK1 by immunoprecipitation (Fig. 6B). TRIM11-RB did not interact with TBK1, indicating that the CC domain of TRIM11 is required for interaction with TBK1. However, TRIM11-RBCC showed much stronger interaction with TBK1 than did TRIM11-FL, indicating that the B30.2/SPRY domain of TRIM11 plays an inhibitory role in this interaction. Next, we tested whether this interaction affects TBK1 activity. TRIM11 domain-deletion constructs were cotransfected with MAVS (Fig. 6C, middle panel) or TBK1 (Fig. 6C, right panel) together with an IFNβ-Luc reporter plasmid in 293T cells. TRIM11-RB lost the ability to inhibit IFNβ promoter activity, whereas TRIM11-RBCC retained inhibitory activity (Fig. 6C), suggesting that the physical interaction determines functional inhibition. To examine which domain of TBK1 is required for the interaction with TRIM11, we performed immunoprecipitation assays using TBK1 domain-deletion mutants (Fig. 6D). Full-length TBK1 (TBK1-FL) and ULD- and CC1 domain-deleted TBK1 constructs showed clear interactions with TRIM11, whereas a CC2 domain-deleted TBK1 construct did not (Fig. 6E). These results indicate that the CC domain of TRIM11 and CC2 domain of TBK1 are necessary for the interaction of these two proteins. It has also been reported that the CC2 domain of TBK1 is required for interaction with the three TBK1 adaptor proteins, NAP1, SINTBAD and TANK [39]. Because TRIM11 and these adaptors share the TBK1-binding CC2 domain, we speculated that TRIM11 competed with these adaptor proteins for the binding to TBK1. To test this, we examined the interaction between TBK1 and TRIM11 in the presence or absence of adaptor proteins by immunoprecipitation assay (Fig. 7). Surprisingly, TRIM11 bound much more tightly to TBK1 in the presence of any of the adaptors than in the absence of adaptor protein (Fig. 7, uppermost two panels). TRIM11 also bound to all three adaptor proteins with different binding affinities (Fig. 7, 3rd panel). One possible explanation for this outcome is that TRIM11-TBK1 interactions are favored by the more physiological state of TBK1 in complex with adaptors. Consistent with the results shown in Figure 6B, the TRIM11-RBCC construct interacted more strongly with TBK1 than did TRIM11-FL, confirming the inhibitory function of the B30.2/SPRY domain in regulating the interaction between TRIM11 and TBK1. Taken together, these results suggest that TRIM11 inhibits RIG-I-mediated IFNβ signaling by association with TBK1 complexes containing adaptor proteins.

### TRIM11 Inhibits IFNβ-dependent Antiviral Response

**Figure 8:**
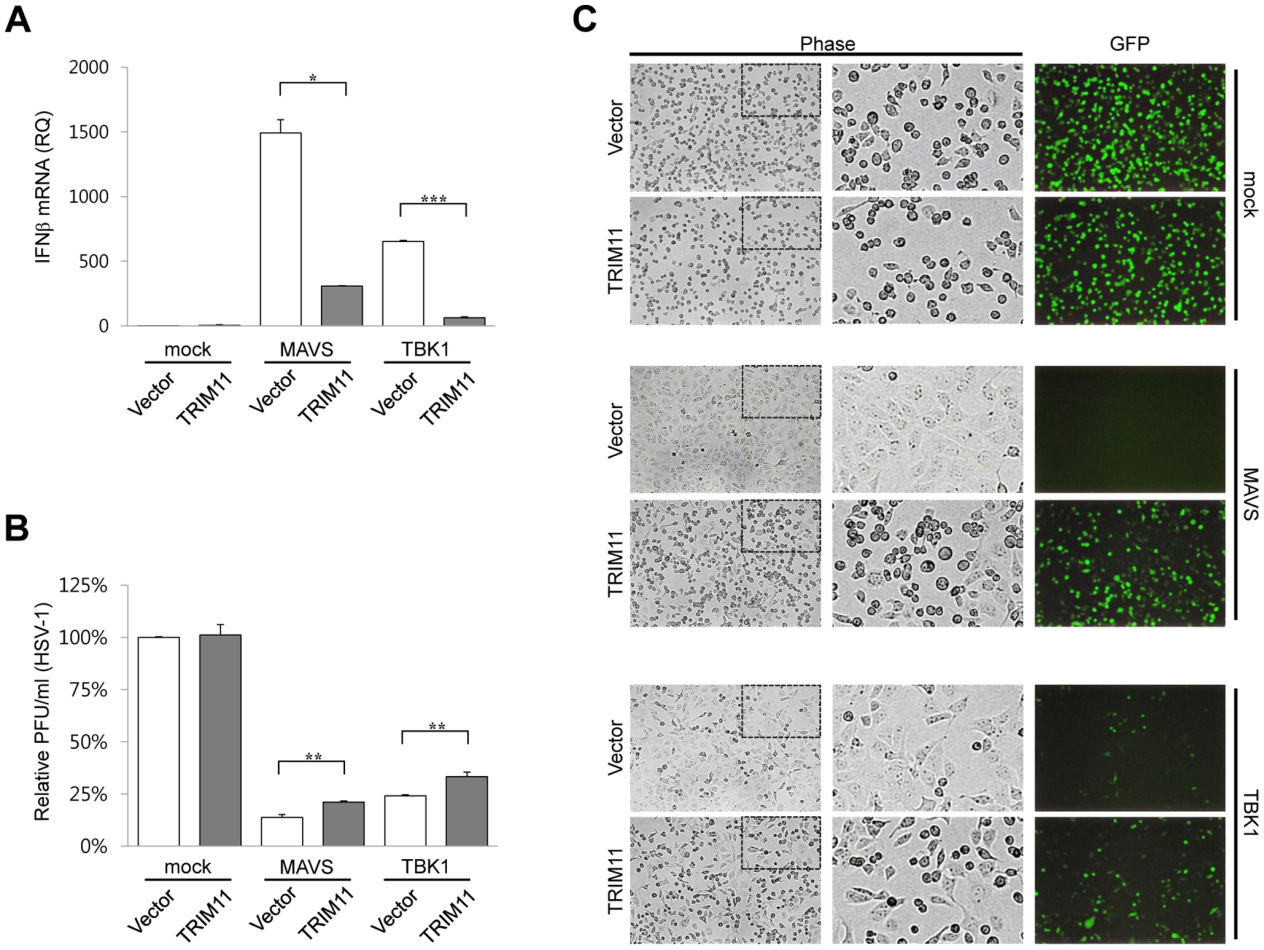
Effect of ectopic expression of TRIM11 on cellular antiviral activity. (**A**) 293T cells were transfected with HA-TRIM11 or empty vector plasmid along with MAVS, TBK1 or empty vector plasmid. After 24 h, supernatants were harvested for treatment to Vero cells and total RNA was extracted and treated with DNase I. Relative quantity (RQ) of IFNβ mRNA was measured by quantitative RT-PCR. (**B**) Vero cells were pretreated with culture supernatants obtained from (A), and then infected with HSV-1. HSV-1 plaques formed in Vero cells were counted by crystal violet staining. (**C**) Vero cells pretreated with the same culture supernatants used in (B) were infected with VSV-GFP (MOI = 10). After 11 h of infection, CPE and GFP fluorescence were analyzed by phase-contrast and fluorescence microscopy. *P<0.05, **P<0.01 ***P<0.001. Box shows zoomed areas.

**Figure 9:**
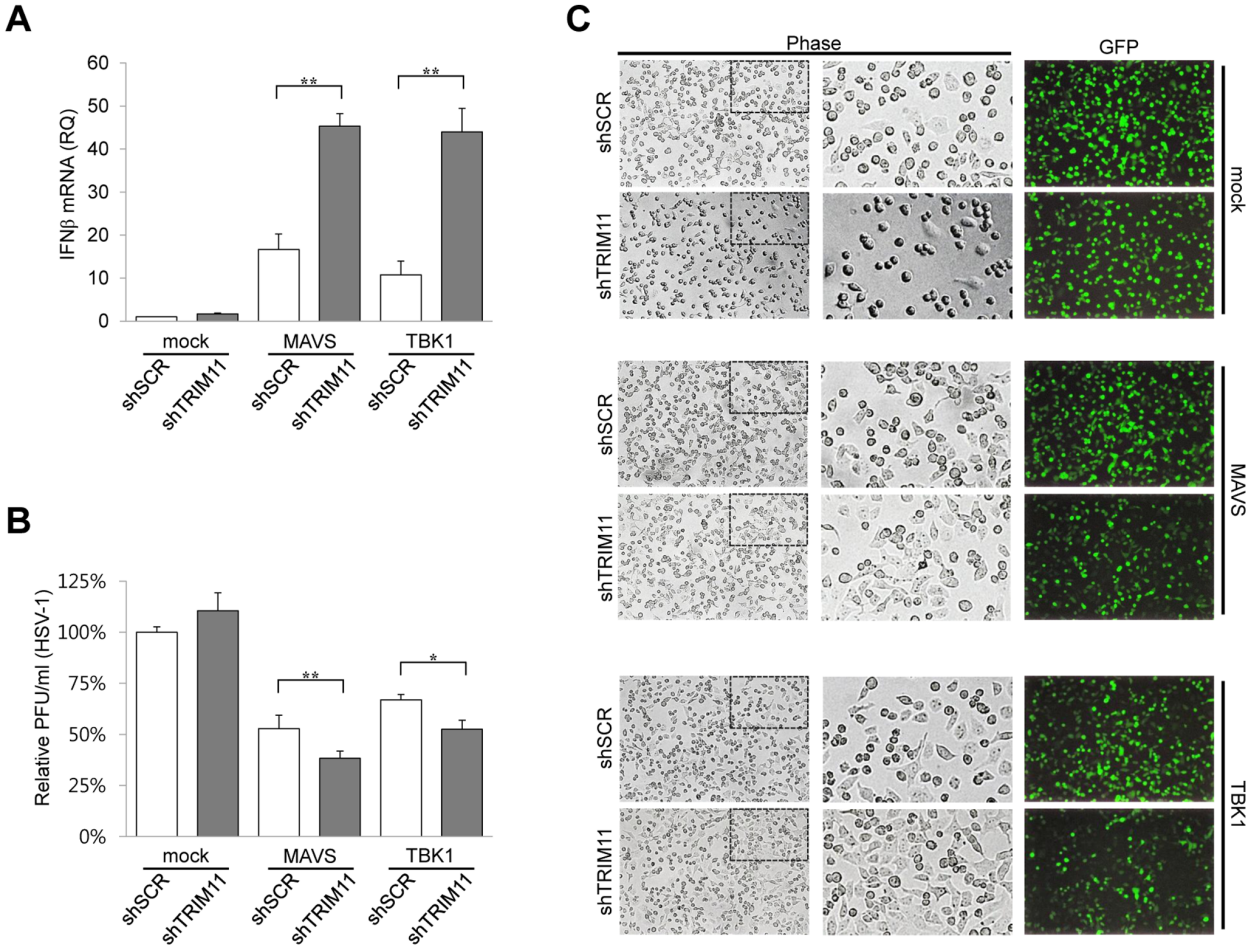
Effect of TRIM11-knockdown on cellular antiviral activity. (**A**) 293T cells stably expressing shTRIM11 or shSCR were transfected with MAVS, TBK1 or empty vector plasmid. After 24 h, supernatants were harvested for treatment to Vero cells and total RNA was extracted and treated with DNase I. Relative quantity (RQ) of IFNβ mRNA measured by quantitative RT-PCR. (**B**) Vero cells were pretreated with culture supernatants obtained from (A) and then infected with HSV-1. HSV-1 plaques formed in Vero cells were counted by crystal violet staining. (**C**) Vero cells pretreated with the same culture supernatants used in (B) were infected with VSV-GFP (MOI = 10). After 11 h of infection, CPE and GFP fluorescence were analyzed by phase-contrast and fluorescence microscopy. *P<0.05, **P<0.01. Box shows zoomed areas.

Because TRIM11 negatively regulated IFNβ expression, we tested the effect of TRIM11 on viral infectivity. IFNβ-containing culture media were obtained from 293T cells transfected with a RIG-I signaling component (MAVS or TBK1) and TRIM11 or empty vector, and then used to treat Vero cells prior to viral infection with HSV-1 or VSV-GFP. The amount of IFNβ mRNA in transfected 293T cells was determined by quantitative RT-PCR. Consistently, IFNβ mRNA increase in MAVS- and TBK1-transfected cells was declined by TRIM11 coexpression (Fig. 8A). Infectivity of HSV-1 was measured by plaque-reduction assays, and infectivity of VSV-GFP was monitored by fluorescence microscopy. The culture supernatants from 293T cells expressing RIG-I signaling components reduced HSV-1 plaque formation (Fig. 8B) and VSV-GFP fluorescence (Fig. 8C) in Vero cells. This antiviral activity was decreased when culture supernatants were prepared from TRIM11-coexpressing cells (Fig. 8). Moreover, a more prominent cytopathic effect (CPE) of virus (rounding and detachment of cells) was observed in cells treated with culture supernatants from TRIM11-overexpressing cell than control cells (Fig. 8C left and middle). To further confirm the effect of TRIM11 on viral infectivity, we tested culture supernatants from TRIM11-knockdown cells in HSV-1 and VSV-GFP infection assays (Fig. 9). As expected, treatment with culture supernatants from TRIM11-knockdown cells more efficiently prevented HSV-1 and VSV-GFP infection than those from control cells. Cumulatively, these data suggest that TRIM11 is a negative regulator of a TBK1-containing signaling pathway leading to IFNβ expression; this action of TRIM11 subsequently inhibits the establishment of an antiviral state in cells.

## Discussion

TRIM11 belongs to the TRIM family, whose members are involved in a broad range of biological processes, such as cell proliferation, differentiation, oncogenesis and apoptosis [22], [23]. Recently, several TRIM family members have emerged as regulators of innate immune responses [21], [24], [40]. It has been reported that TRIM21 is needed for polyubiquitination and degradation of IRF3 and IRF7 [41], [42], and TRIM28 mediates SUMOylation of IRF7 [43]. TRIM25 has been shown to induce Lys63-linked ubiquitination of RIG-I [36]. And TRIM38 promotes the ubiquitination and degradation of NAP1 [44]. In addition to their roles in innate immunity, many TRIM proteins are involved in diverse physiological processes by virtue of their regulation of the turnover and activity of target proteins through ubiquitination [22]. TRIM11 mediates ubiquitination of Humanin, ARC105, PAX6, and PHOX2B, and thereby promotes their proteasomal degradation [25]–[28]. However, although TRIM11 clearly bound TBK1 and inhibited its downstream signaling, we found no evidence for TRIM11-mediated TBK1 ubiquitination in this study (Figure S2A). Consistent with this, deletion of the RING domain (the catalytic domain responsible for E3 ubiquitin ligase activity) did not affect TRIM11 inhibition of IFNβ expression (Figure S2C). Therefore, we conclude that the E3 ligase activity of TRIM11 is not necessary for its role in IFNβ regulation.

TRIM11 inhibited IRF3 activation and led to a decrease in the production of IFNβ through binding to the CC2 domain of TBK1, a domain to which the TBK1 adaptor proteins NAP1, TANK, and SINTBAD also bind [39]. An interesting aspect of this molecular interaction is that, rather than competing with TRIM11 for TBK1, these adaptor proteins enhanced the strength of TRIM11-TBK1 binding. Also interesting is the observation that TRIM11-TBK1 binding was much stronger in the absence of the C-terminal B30.2/SPRY domain of TRIM11. Further research is required to elucidate the detailed mechanism of these salient features of the molecular interaction, especially with respect to the protein complex structure.

In this study, we show that TRIM11 inhibited the activation of IRF3 and ultimately led to a reduction in IFNβ production. Although TRIM11 interacts with a TBK1 complex containing adaptor proteins, the exact molecular mechanism by which TRIM11 inhibits TBK1 activity has yet to be determined. In vitro kinase assay using immunoprecipitated proteins (TBK1, IRF3, and TRIM11) revealed that TRIM11 does not directly inhibit the kinase activity of TBK1 (Figure S3). One appealing idea is that TRIM11 prevents interactions between the TBK1 complex and other molecules required for TBK1 activation, such as MAVS, TRAF3, and possibly other unknown proteins.

Because aberrant production of IFNβ is related to inflammatory and autoimmune diseases, IFNβ production is tightly regulated [10]. TBK1 activation, in particular, must be tightly controlled to prevent excessive harmful immune responses, because a number of signals initiated by diverse PRRs converge on TBK1 activation to promote IFNβ production. Recent studies have reported that a number of proteins negatively regulate IFNβ by targeting specific elements in the pathway that leads to its production. For example, NLRX1 (NOD-like receptor X1), PCBP2 (poly(C)-binding protein 2), and PSMA7 target MAVS [14], [45], [46], SIKE (suppressor of IKK-epsilon), SHIP-1 (Src homology 2 domain-containing inositol-5-phosphatase-1), NLRP4 and TRIP target for TBK1 [16], [17], [47], [48], TRIM38 target for NAP1 [44], and Pin1 (peptidyl-prolyl cis/trans isomerase NIMA-interacting 1), TRIM21 and the v-Maf oncogene homolog MafB target IRF3 [41], [49], [50]. Here we provide additional insight into the negative regulation of IFNβ production, reporting a novel function of TRIM11 targeting TBK1.

## Supporting Information

Figure S1 **Effect of TRIM11 on TOP-flash promoter activity.** 293T cells were cotransfected with GFP-β-catenin (S45Y) plasmid together with TOP-flash reporter and CMV-β-gal with increasing amount of HA-TRIM11 plasmid. After 36 h, the luciferase activity was measured and normalized for transfection efficiency using β-gal activity. Results are mean values from three independent experiments. Error bar represents SD. Expression levels of HA-TRIM11 were assessed by anti-HA immunoblotting (inset).
(PDF)

Figure S2 **Inhibitory role of TRIM11 in IFNβ production is independent of RING domain, which is essential for its E3 ligase activity.** (**A**) FLAG-TBK1 and Ubi-His plasmid were transiently cotransfected with HA-TRIM11 or empty vector into 293T cells. After 36 h, cells were lysed and immunoprecipitated with anti-FLAG agarose. Immunoprecipitates were analyzed by immunoblotting with the anti-TBK1, anti-FLAG and anti-HA antibodies. (**B**) Schematic representation of TRIM11 full-length (FL) and RING domain-deleted mutant (ΔRING). (**C**) 293T cells were cotransfected with TBK1 plasmid and TRIM11 (FL or ΔRING) plasmid together with IFNβ-Luc and CMV-β-gal plasmid. After 36 h, the luciferase activity was measured and normalized for transfection efficiency using β-gal activity. Results are mean values from three independent experiments. Error bar represents SD.
(PDF)

Figure S3 **TRIM11 does not directly inhibit TBK1 kinase activity.** For in vitro kinase assay, 293T cells were separately transfected with FLAG-TBK1, FLAG-IRF3 and HA-TRIM11 plasmid. After 36 h, cells were lysed and immunoprecipitated with anti-FLAG agarose for TBK1 and IRF3 or anti-HA agarose for TRIM11. Immunoprecipitated kinases (TBK1) and substrate (IRF3) were incubated with increasing amount of TRIM11 in kinase reaction buffer (20 mM HEPES pH 7.5, 10 mM MgCl_2_, 10 mM p-nitrophenyl phosphate, 1 mM DTT, 0.1 mM Na_3_VO_4_, 1 mM ATP) for 30 min at 30°C. Reaction mixture was resolved by SDS-PAGE and analyzed by immunoblotting with anti-phospho-IRF3, anti-TBK1, anti-IRF3, and anti-TRIM11 antibodies.
(PDF)
