# Supplementary figures and images for: TRIM11 Negatively Regulates IFNβ Production and Antiviral Activity by Targeting TBK1
Source: PLoS One. 2013 May 13;8(5):e63255. doi: 10.1371/journal.pone.0063255 (PMC3652858; doi:10.1371/journal.pone.0063255)

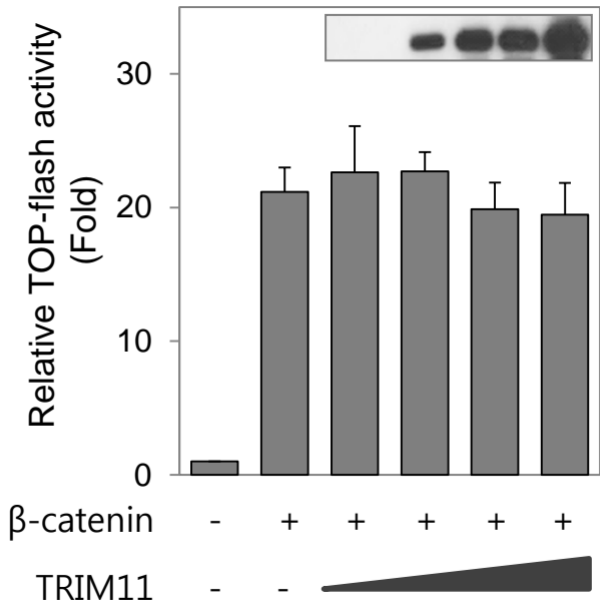

Supplement: Figure S1 — Effect of TRIM11 on TOP-flash promoter activity. 293T cells were cotransfected with GFP-β-catenin (S45Y) plasmid together with TOP-flash reporter and CMV-β-gal with increasing amount of HA-TRIM11 plasmid. After 36 h, the luciferase activity was measured and normalized for transfection efficiency using β-gal activity. Results are mean values from three independent experiments. Error bar represents SD. Expression levels of HA-TRIM11 were assessed by anti-HA immunoblotting (inset). (PDF) [file pone.0063255.s001.pdf]

**A**

|           |   |   |
|-----------|---|---|
| HA-TRIM11 | - | + |
| FLAG-TBK1 | + | + |
| Ubi-His   | + | + |

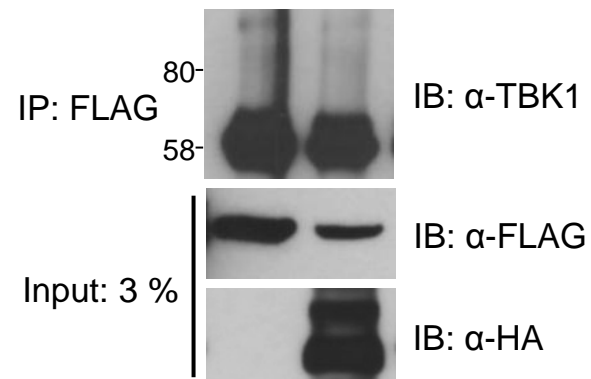**B**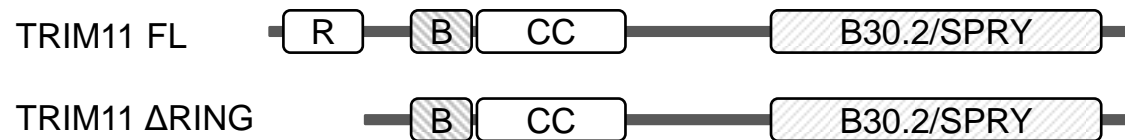**C**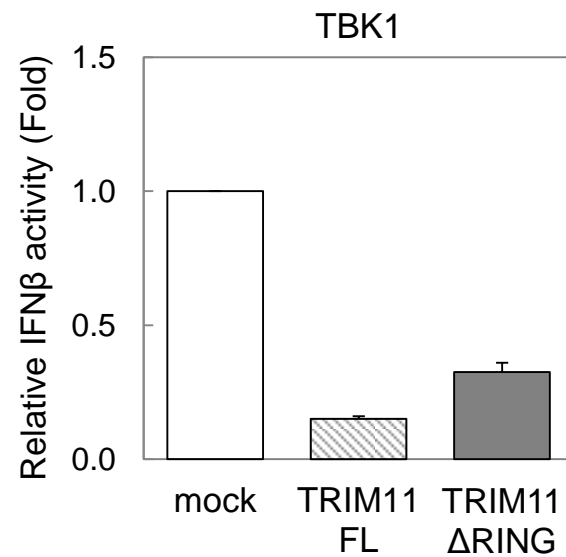

Supplement: Figure S2 — Inhibitory role of TRIM11 in IFNβ production is independent of RING domain, which is essential for its E3 ligase activity. (A) FLAG-TBK1 and Ubi-His plasmid were transiently cotransfected with HA-TRIM11 or empty vector into 293T cells. After 36 h, cells were lysed and immunoprecipitated with anti-FLAG agarose. Immunoprecipitates were analyzed by immunoblotting with the anti-TBK1, anti-FLAG and anti-HA antibodies. (B) Schematic representation of TRIM11 full-length (FL) and RING domain-deleted mutant (ΔRING). (C) 293T cells were cotransfected with TBK1 plasmid and TRIM11 (FL or ΔRING) plasmid together with IFNβ-Luc and CMV-β-gal plasmid. After 36 h, the luciferase activity was measured and normalized for transfection efficiency using β-gal activity. Results are mean values from three independent experiments. Error bar represents SD. (PDF) [file pone.0063255.s002.pdf]

|           |   |   |   |   |                                                                                     |   |   |
|-----------|---|---|---|---|-------------------------------------------------------------------------------------|---|---|
| ATP       | + | + | - | + | +                                                                                   | + | + |
| FLAG-TBK1 | + | - | + | + | +                                                                                   | + | + |
| FLAG-IRF3 | - | + | + | + | +                                                                                   | + | + |
| HA-TRIM11 | - | - | - | - | 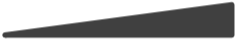 |   |   |

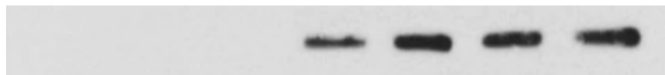

IB:  $\alpha$  - p-IRF3

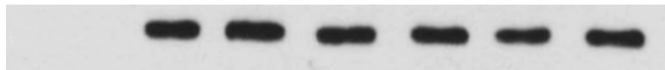

IB:  $\alpha$  -IRF3

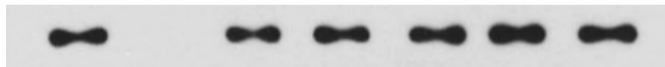

IB:  $\alpha$  - TBK1

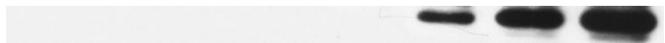

IB:  $\alpha$  - TRIM11

Supplement: Figure S3 — TRIM11 does not directly inhibit TBK1 kinase activity. For in vitro kinase assay, 293T cells were separately transfected with FLAG-TBK1, FLAG-IRF3 and HA-TRIM11 plasmid. After 36 h, cells were lysed and immunoprecipitated with anti-FLAG agarose for TBK1 and IRF3 or anti-HA agarose for TRIM11. Immunoprecipitated kinases (TBK1) and substrate (IRF3) were incubated with increasing amount of TRIM11 in kinase reaction buffer (20 mM HEPES pH 7.5, 10 mM MgCl2, 10 mM p-nitrophenyl phosphate, 1 mM DTT, 0.1 mM Na3VO4, 1 mM ATP) for 30 min at 30°C. Reaction mixture was resolved by SDS-PAGE and analyzed by immunoblotting with anti-phospho-IRF3, anti-TBK1, anti-IRF3, and anti-TRIM11 antibodies. (PDF) [file pone.0063255.s003.pdf]
